# Assessing Electrocardiogram and Respiratory Signal Quality of a Wearable Device (SensEcho): Semisupervised Machine Learning-Based Validation Study

**DOI:** 10.2196/25415

**Published:** 2021-08-12

**Authors:** Haoran Xu, Wei Yan, Ke Lan, Chenbin Ma, Di Wu, Anshuo Wu, Zhicheng Yang, Jiachen Wang, Yaning Zang, Muyang Yan, Zhengbo Zhang

**Affiliations:** 1 Medical School of Chinese PLA Beijing China; 2 Department of Hyperbaric Oxygen The First Medical Center Chinese PLA General Hospital Beijing China; 3 Beijing SensEcho Science & Technology Co., Ltd. Beijing China; 4 School of Biological Science and Medical Engineering Beihang University Beijing China; 5 University of Washington Seattle, WA United States; 6 PAII Inc. Palo Alto, CA United States; 7 Department of Kinesiology Shanghai University of Sport Shanghai China; 8 Centre for Artificial Intelligence in Medicine Medical Innovation Research Department Chinese PLA General Hospital Beijing China

**Keywords:** signal quality, electrocardiogram, respiratory signal, isolation forest, machine learning, mobile health

## Abstract

**Background:**

With the development and promotion of wearable devices and their mobile health (mHealth) apps, physiological signals have become a research hotspot. However, noise is complex in signals obtained from daily lives, making it difficult to analyze the signals automatically and resulting in a high false alarm rate. At present, screening out the high-quality segments of the signals from huge-volume data with few labels remains a problem. Signal quality assessment (SQA) is essential and is able to advance the valuable information mining of signals.

**Objective:**

The aims of this study were to design an SQA algorithm based on the unsupervised isolation forest model to classify the signal quality into 3 grades: good, acceptable, and unacceptable; validate the algorithm on labeled data sets; and apply the algorithm on real-world data to evaluate its efficacy.

**Methods:**

Data used in this study were collected by a wearable device (SensEcho) from healthy individuals and patients. The observation windows for electrocardiogram (ECG) and respiratory signals were 10 and 30 seconds, respectively. In the experimental procedure, the unlabeled training set was used to train the models. The validation and test sets were labeled according to preset criteria and used to evaluate the classification performance quantitatively. The validation set consisted of 3460 and 2086 windows of ECG and respiratory signals, respectively, whereas the test set was made up of 4686 and 3341 windows of signals, respectively. The algorithm was also compared with self-organizing maps (SOMs) and 4 classic supervised models (logistic regression, random forest, support vector machine, and extreme gradient boosting). One case validation was illustrated to show the application effect. The algorithm was then applied to 1144 cases of ECG signals collected from patients and the detected arrhythmia false alarms were calculated.

**Results:**

The quantitative results showed that the ECG SQA model achieved 94.97% and 95.58% accuracy on the validation and test sets, respectively, whereas the respiratory SQA model achieved 81.06% and 86.20% accuracy on the validation and test sets, respectively. The algorithm was superior to SOM and achieved moderate performance when compared with the supervised models. The example case showed that the algorithm was able to correctly classify the signal quality even when there were complex pathological changes in the signals. The algorithm application results indicated that some specific types of arrhythmia false alarms such as tachycardia, atrial premature beat, and ventricular premature beat could be significantly reduced with the help of the algorithm.

**Conclusions:**

This study verified the feasibility of applying the anomaly detection unsupervised model to SQA. The application scenarios include reducing the false alarm rate of the device and selecting signal segments that can be used for further research.

## Introduction

### Background

Wearable devices have been widely adopted for daily health care monitoring during the past decades. Many researchers utilize wearable sensors to continuously monitor physiological signals for mobile health (mHealth) and ubiquitous health (uHealth) app studies [1-3]. Recently, wearable devices have shown their potential in providing early warning of disease deterioration, chronic disease self-management, rehabilitation assessment, among others [4-7]. For example, some clinical deterioration changes in physiological signals could be often present 8-24 hours before a severe life-threatening event such as an unplanned intensive care unit admission or sudden cardiac death [8,9]. In these scenarios, signal quality is essential to acquire the valuable information from the time-series physiological signals which are very sensitive to noise. Signal quality assessment (SQA) facilitates reducing the high false alarm rate caused by signal quality [10] and can be applied to automatically screen the “real-world” data for further research. However, SQA of wearable physiological signals has not been well investigated. Such inadequate studies on signal quality reliability limit the further clinical deployment of these devices in the medical sector [11]. Therefore, it is important to develop a feasible method to evaluate the signal quality from wearable physiological monitoring systems and SQA is one of the basics of mHealth research and apps.

### Related Work

It is widely recognized that the electrocardiogram (ECG) and respiratory signals are crucial for both patient monitoring and health status identification, and thus are being extensively investigated. Various solutions have been proposed to accomplish ECG SQA [12,13]. Some early studies, such as those by Langley et al [14] and Johannesen [15], considered the poor quality of ECG signals when their waveform features exceed the preset thresholds [16]. Several signal quality indices (SQIs) such as kSQI (the kurtosis of the distribution), sSQI (the skewness of the distribution), and pSQI (the relative power in the QRS complex) were introduced [17-19], which use the features from the time domain and the frequency domain of the ECG signals to assess the quality [20]. Another approach to ECG SQA is based on template matching. Researchers usually compare the similarity between the signals and a template that is fixed or derived from historical data [21]. In recent years, leveraging the machine learning technology in the medical domain, many researchers used the time–frequency domain features and SQIs to build machine learning models to achieve ECG SQA [16,21-23]. For example, Zhao et al [23] provided an algorithm based on convolutional neural networks, which aimed at identifying noisy segments from wearable ECG recordings. Zhang et al [16] compared the performance of random forest (RF), support vector machine (SVM), and their variants for ECG SQA with nonlinear features. For respiratory signals, Charlton et al [24] developed an SQI for the impedance pneumography respiratory signal by using the breath duration variations and by examining whether the peaks and troughs are clear and similarity of breath morphologies. However, research on respiratory SQA remains in its infancy. Few studies have investigated this topic so far to our knowledge.

### Challenges

Owing to the rapid development of wearable devices, there is an explosion of the volume of data being acquired and available for research studies. However, the importance of the SQA process has been underestimated. The limitations of previous studies and the challenges we are currently facing are summarized as follows: For ECG SQA, first, signal quality is often judged subjectively, which lacks objective quantitative criteria, and the standard of signal quality was relatively fuzzy in previous studies [25,26]. Second, most of the SQAs were conducted under well-designed laboratory conditions by using simulated signals [27], or assessed the signals from bedside monitors. Thus, signals are highly different from those measured by wearable devices in daily lives because the noise in the laboratory was relatively single and controllable, or the signal quality was good for most of the time. Third, although most of the methods have good performances on ECG SQA, the dominant methods are still supervised machine learning models [16]. There is a concern that these models are at a high risk of overfitting, leading to unsatisfying model generalization. Moreover, when using supervised models, it is quite challenging to prepare tons of labeled data and even impossible for each research group to use the fixed open-source data sets, such as the MIT-BIH Arrhythmia Database (MITDB), to build models, which were not built for SQAs. In addition, hardware designs of wearable devices are diverse, resulting in aggravating incomplete generalization of data and poor migration performance of models. One possible solution to this problem is to build dedicated models using specific wearable devices and the data they collected. For respiratory SQAs, the challenge lies in the various respiratory patterns. Compared with ECG signals, respiratory signals have more diverse forms, broader spectral distribution, and different noise sources.

### Study Objectives

To address the above problems, we pioneered the idea that the SQA process can be seen as an anomaly detection. The basic hypothesis of our study was that the decline of the signal quality can be quantified with the increase of the anomaly and can be detected by the machine learning model. The application scenarios we expected of the algorithm include reducing the false alarms caused by poor signal quality and selecting the high-quality signal segments for further research. The objectives and main components of this paper are to:

- design an algorithm based on the unsupervised machine learning model, isolation forest (IF), to classify the ECG and respiration signal quality into 3 different grades: good, acceptable, and unacceptable.
- quantitatively evaluate the performance of the algorithm on a small amount of labeled data. Further validation of the algorithm was implemented on several cases of data to prove its feasibility.
- apply the SQA algorithm to real-world data to demonstrate that the algorithm has the potential to reduce the false alarms caused by poor signal quality.

## Methods

### The Wearable Device and Data Sources

**Figure 1:**
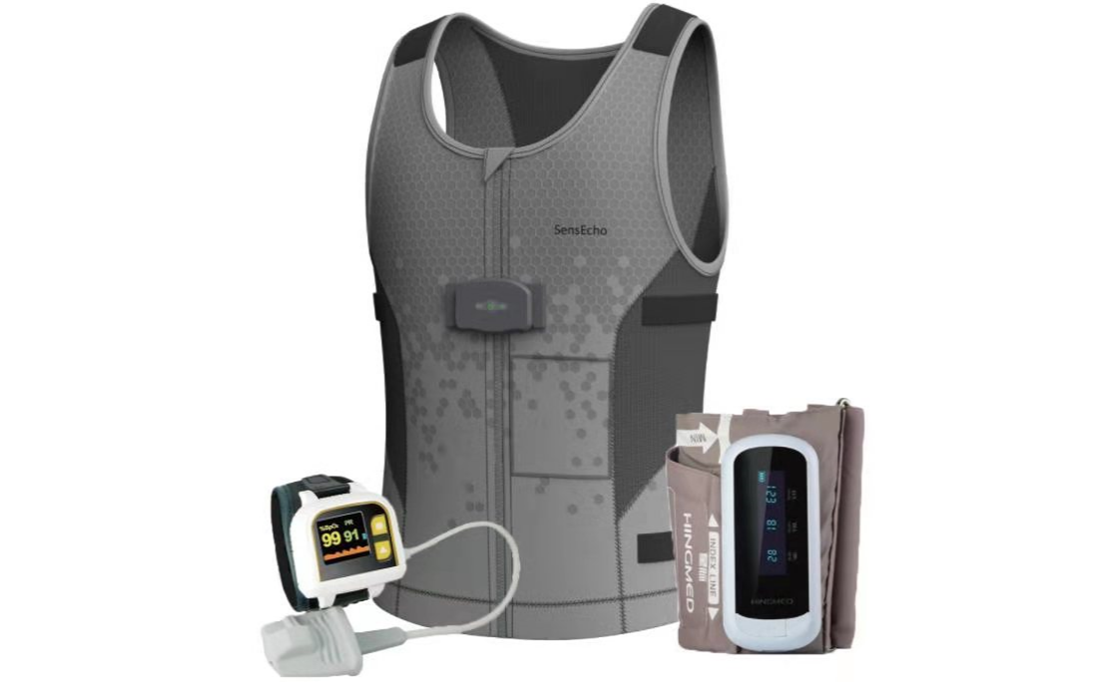
Picture of SensEcho, including third-party oximeter and cuff blood pressure monitor.

The medical-grade wearable device we used was a self-developed physiological signal monitoring system, SensEcho (Figure 1) [28], which has received clearance from the China Food and Drug Administration (CFDA) and has been deployed in the general wards of the Hyperbaric Oxygen (HBO) Department in Chinese PLA General Hospital (PLAGH) since 2018. The core wearable device of SensEcho is a vest, which provides a single-lead ECG signal, chest and abdominal respiratory signals via the respiratory inductive plethysmography (RIP) technology, and triaxial acceleration signals. It also allows for communication with other third-party wearable devices such as oximeters and blood pressure monitors. Its battery supports continuous monitoring for a minimum of 24 hours. For detailed information about SensEcho and the monitoring system, please refer to [29]. At the time of writing, SensEcho has collected more than 1000 records from patients and healthy individuals. Each record contains nearly 24-hour physiological signal monitoring results; thus, a large pool of data is available for research purposes. Data collection was carried out in a clinical environment for patients and from daily lives for healthy individuals without restriction of movement and activity. In this study, we used the single-lead ECG signal and chest respiration signal from the data pool to establish and evaluate the algorithm. This study was approved by the ethics committee of PLAGH (No. S2018-095-01).

### Signal Quality Classification

#### Overview

The definition of signal quality was indistinct in previous studies, but some of the studies have proposed a few quantitative criteria. Inspired by [26] and the results of our pre-experiment, 10- and 30-second segments of ECG and respiratory signals were considered sufficient for our study. In early SQA studies, 5 quality groups (excellent, good, adequate, poor, and unacceptable) [15], 3 quality groups (acceptable, indeterminate, and unacceptable) [18,30,31], and 2 quality groups (acceptable and unacceptable) [32-35] were investigated. Based on previous studies, we defined 3 grades of signal quality for different requirements: (1) *good signal quality* refers to that in which the signal waves are clear, and signal of this grade can be analyzed automatically in follow-up studies and have confidence high enough for waveform feature analysis; (2) *acceptable signal quality* refers to that in which the R peak in ECG signal and peaks and troughs of respiratory signal can be accurately located by the algorithm, and the signal of this grade can be used for relative accurate heart rate and respiratory rate analysis. In addition, this grade is often the most difficult to distinguish and the signal availability depends on the specific apps where further manual determination might be needed; (3) *unacceptable signal quality* refers to that in which the waveform in the window is chaotic, and this grade of signal should be dropped because of the unreliable results obtained in signal analysis.

A brief description of characteristics of signal noise sources and their patterns is summarized in the following subsections [12,22,36,37].

#### Baseline Wander

ECG signals are affected by respiratory motion, body movement, and poor electrode contact. Respiratory signals are more sensitive to movement and breath pattern than ECG signals. One final major expression in signals is different levels of baseline wander.

#### High-Frequency Noise

For ECG signals, high-frequency noise usually includes power line interference, myoelectricity interference, and movement artifact. For respiratory signals measured by the RIP, the noise often is from vibrations caused by movement, such as moving or speaking.

#### Signal Loss

This is also a pervasive pattern in daily signal acquisition, which usually appears as a straight line. Based on the noise source and expression analysis, the quantitative evaluation criteria defined by clinical and engineering experts in our study are listed in Table 1.

**Table 1:** Quantitative signal quality assessment criteria.

Quality grade	Electrocardiogram	Respiratory signal
Good	ECG rhythm is clear; each QRS waveform can be distinguished with naked eyes. No signal loss in the observation window. Maximal baseline wander amplitude is less than one-third of signal amplitude in the observation window. Pathological changes do not influence the signal quality assessment; the recognized obvious pathological patterns can be classified as good quality, such as ventricular premature beats.	Regular waveform lasts for more than three-fourth of the observation window. Maximal baseline wander amplitude is less than the signal amplitude in the observation window. High-frequency noise can be easily filtered and does not affect the judgment of the respiratory signal waveform.
Acceptable	Low-intensity high-frequency noise; the R waves in signal can be recognized accurately. No more than 2 high-frequency impulse noises occur in the observation window. Less than 2-second signal loss in the observation window. The maximal baseline wander amplitude is below the signal amplitude. Fewer than 2 cardiac cycles in which the QRS waves cannot be recognized are allowed.	One-half to one-fourth of the signal is clear; respiratory rhythm can be identified. Time for signal loss or hold breath lasts less than one-half of the observation window. High-frequency noise has only a little impact on the judgment of the overall waveform trend.
Unacceptable	Full of noise. More than 2 R peaks in the observation window cannot be distinguished. Excessive baseline wander. Signal loss lasts more than 2 seconds. Suspected pathological changes, but the cause is not clear.	The pattern of respiratory waveform is difficult to recognize. Severe baseline wander.

### Isolation Forest

IF is an unsupervised anomaly detection model that has been applied to many fields such as streaming data processing and mineral mapping [38,39]. IF grows an ensemble of binary trees to estimate the degree of being an anomaly of an instance. As anomalies are more susceptible to isolation, they have a short path length [38,40]. Furthermore, an anomaly score can be obtained by measuring/estimating the average path height of the ensemble of binary trees (in [40], the authors named them iTree). The IF model is based on 2 fundamental assumptions and premises. The first one is that the anomalies should be “few and different.” If a pattern occurs frequently in the training set, it will be more likely to be perceived as normality, although it is indeed an anomaly manually determined. The second one is that the training set should conclude as many normal patterns of the signals as possible. It is necessary to guarantee that the training set has a large enough variety, especially for normal signals; otherwise the model will be more likely to classify a brand-new pattern as an anomaly.

**Figure 2:**
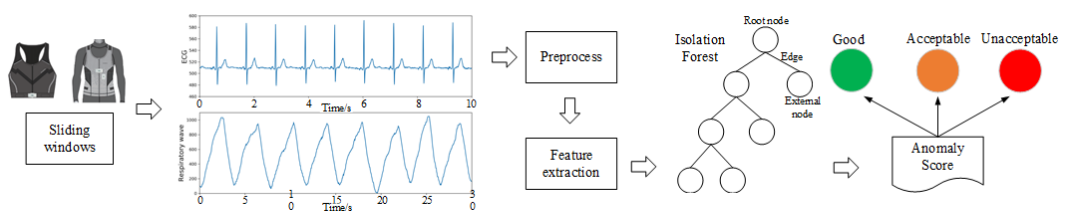
General framework of the signal quality assessment algorithm for the electrocardiogram and respiratory signal.

Based on the above theory, the general framework of the SQA algorithm is shown in Figure 2. We built models for ECG and respiratory SQA, respectively, and both models were trained and evaluated independently. The preprocess included filtering, removing the outliers, removing the baseline, and normalization. We then selected 8 and 18 features from the time and frequency domains of the ECG and respiratory signals, respectively. Skewness, kurtosis, and distances of adjacent waveforms calculated using the dynamic time warping method [41] were the key features we used, which also have been widely adopted as the key variables to construct the SQIs [17,18,42,43]. The skewness and kurtosis are defined as Equations (1) and (2). Other features we used in this study were the features from amplitude of the signal in the time–frequency domain, power spectrum distribution, and power spectral density.


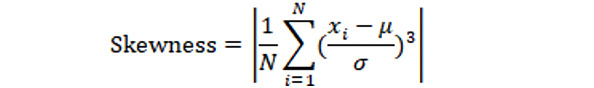
 (1)


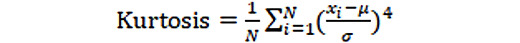
 (2)

where *N* is the sample points of the signal, is the mean value, and σ is the SD.

### Experiment Design

#### Overview

The experimental process involved 4 key steps. The model training and validation were conducted on 4 nonoverlapping data sets extracted from the sizable volume data pool and possessed different functions: (1) training set, which was used to train the IF model; (2) validation set, which was used to find the thresholds that map the anomaly scores obtained by the model to the triclassification SQA results; (3) test set, which was used to quantitatively measure the generalization ability of the model; and (4) case set, which was used to qualitatively evaluate the model’s performance by feeding a whole case of data to it. Some details of these 4 data sets are specified in the following sections.

#### Training Set

We selected a set of 24-hour monitoring records which met the following inclusion criteria: (1) signal acquisition was stable by manual determination; (2) no signal loss for extended periods (over 10 minutes) during monitoring; and (3) no persistent atrial fibrillation during monitoring. Based on these, 30 records were included and we selected 3-10 of them randomly to construct the training set with their whole data. We repeated the selection process 20 times for each epoch, that is, we randomly selected 3 records to construct the training set 20 times to find the best performance of the model.

#### Validation Set

We used the data from 16 patients and 8 healthy individuals to construct this data set, expecting that the pathological changes were more complex and the proportion of anomaly was relatively high. We selected 10,000 windows of signals from the records and then removed half of them that were obviously of high quality. The data set was labeled independently by 3 pretrained graduate students of biomedical engineering according to the criteria in the above section. To guarantee label accuracy, we used the agreed result to define the final label, and dropped the windows of signals that had conflicting label results. Moreover, we asked clinical specialists to mark whether the ECG signals in the data set were pathological. If pathological manifestations of the signal, such as arrhythmia or ST-segment elevation, were confirmed, the number of this signal segment was recorded additionally. After the manual annotation of the data set is completed, the anomaly scores of the labeled data can be obtained by feeding the signals to the trained SQA model. Then, thresholds T1 and T2 were set to map the anomaly scores to the signal quality grades. We adjusted the values of T1 and T2, respectively, to find the best performance thresholds, which were fixed and used in the next step.

#### Test Set

Test set data came from 8 patients and 9 healthy individuals, because we expected the test set to be somewhat different from the validation set and to be closer to practical use. We extracted 1 window of signals every 6 minutes and this data set initially comprised 5500 windows of signals, which were labeled in the same way as the validation set. We used the T1 and T2 values determined by the validation set to obtain the classification results of the model, and then quantitatively evaluated the generalization ability of the model. The basic information about the individuals involved in the validation and test sets is summarized in Table 2.

**Table 2:** Basic information about the individuals utilized in the validation and test sets.

Characteristics				Validation set				Test set				
				Patients (n=16)		Healthy individuals (n=8)		Patients (n=8)		Healthy individuals (n=9)		
**Demography**												
	Male, n (%)			9 (56)		8 (100)		5 (63)		5 (56)		
	Age (year), mean (Q1-Q3)			56 (52-60)		27 (25-33)		69 (65-73)		32 (27-41)		
	Height (cm), mean (Q1-Q3)			168 (160-170)		174 (171-176)		165 (156-174)		171 (157-175)		
	Weight (kg), mean (Q1-Q3)			68 (55-76)		68 (59-74)		70 (64-78)		73 (58-74)		
**Comorbidity, n (%)**												
		Coronary heart disease	12 (75)		—		5 (63)		—			
		Hyperlipemia	9 (56)		—		4 (50)		—			
		Hypertension	9 (56)		—		7 (88)		—			
		Diabetes	8 (50)		—		4 (50)		—			
		Pulmonary nodule	4 (25)		—		2 (25)		—			
		Sleep apnea syndrome	2 (13)		—		—		—			

#### Case Set

We fed several cases of data to the model. Different grades of signal quality segments were marked in different colors. We looked at several observation windows in detail to determine whether the model classification results were correct. Note that we are particularly concerned about the pathological changes in the cases, because we expected pure pathological changes to be not misclassified as poor signal quality.

### Data Set Descriptions

**Figure 3:**
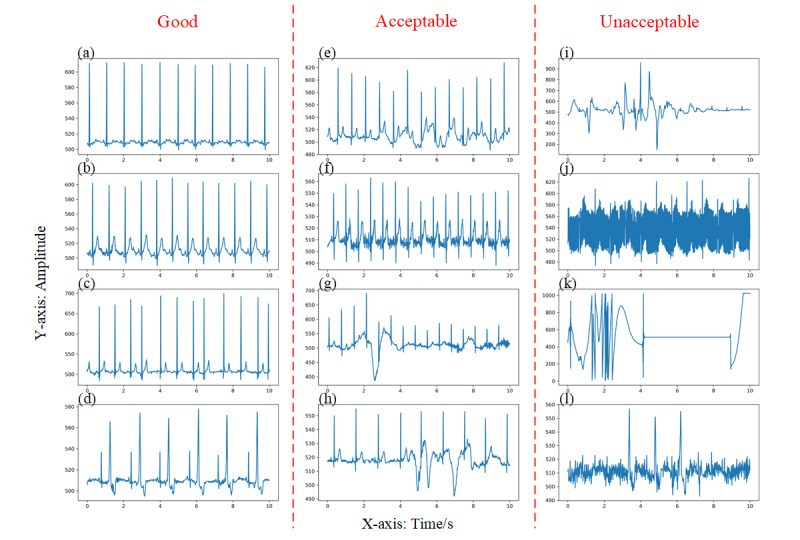
Typical examples of the labeled electrocardiogram signals. (a) & (b) are the normal, good-quality signals; (c) is suspected of arrhythmia while (d) is an expression of ventricular premature beats (VPBs); (e) – (h) show examples of baseline wander, power line interference and impulse noise; (i) – (k) show examples of severe noise and signal loss; (l) is suspected of VPBs but the signal is unclear.

**Figure 4:**
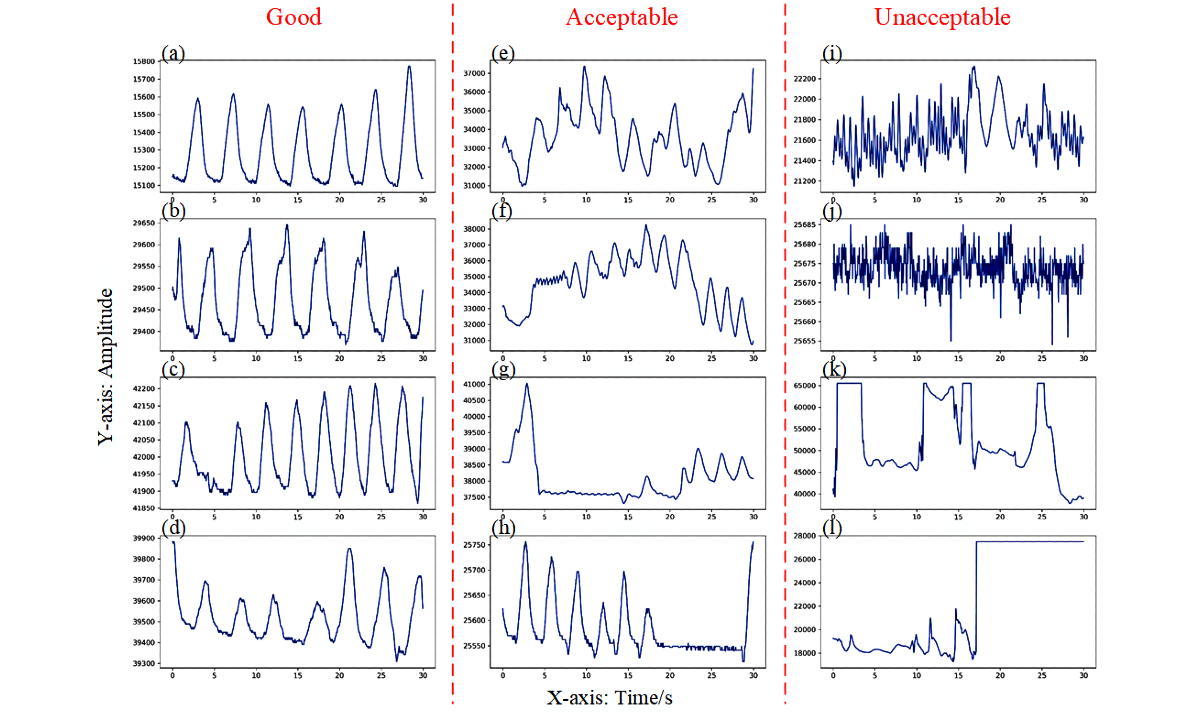
Typical examples of the labeled respiratory signals. (a) – (d) show clear and regular respiratory waves; signals in (e) – (h) do not have enough regularity, apnea occupies some small segments in the observation windows; (i) – (l) show severe noise and signal loss in the observation windows.

After data labeling, we obtained the final validation and test sets. The validation set consisted of 3460 and 2086 ECG and respiratory labels (all agreed), respectively. Of the 3460 ECG labels, 3022 (87.34%) were good, 189 (5.46%) were acceptable, and 249 (7.20%) unacceptable. Of the 2086 respiratory labels, 1308 (62.70%) were good, 511 (24.50%) acceptable, and 267 (12.80%) unacceptable. The test set consisted of 4686 and 3341 ECG and respiratory labels, respectively. Of the 4686 ECG labels, 3767 (80.39%) were good, 284 (6.06%) acceptable, and 635 (13.55%) unacceptable, compared with 2255 (67.49%), 587 (17.57%), and 499 (14.94%), respectively, for respiratory labels. Some typical examples of the labeled ECG and respiratory signals are shown in Figures 3 and 4.

Meanwhile, for the pathological ECG labels, a total of 661/3460 (19.10%) windows of ECG signal in the validation set were marked. Of these, 648 (98.0%) were labeled as having good quality and the rest (13/661, 1.9%) as acceptable quality. In the test set, 634/4686 (13.53%) windows of signal were pathological; of these, 618 (97.5%) were of good quality and the rest (16/634, 2.5%) were of acceptable quality.

### Performance Evaluation

The programming language we used was Python (version 3.6.5) and the major library in this study is scikit-learn (version 0.23.1). The proposed algorithm contained 2000 trees and had 5% anomaly proportion as parameters. We first evaluated the algorithm’s performance according to its accuracy score, which is defined as the number of correctly classified samples divided by the total number of samples. Some additional evaluation indicators included mean precision rate, recall rate, and F1 score (marco-F1). To further evaluate the performance of the algorithm, we compared the algorithm with the self-organizing maps (SOMs) [44] and 4 classical supervised machine learning models, namely, logistic regression (LR), SVM, RF, and extreme gradient boosting (XGB). It should be noted that the SOM is an unsupervised model based on artificial neural network and has been applied in several health care–related signal processing fields such as photoplethysmogram signal classification [45,46] and health situation monitoring [47,48]. The SOM library used in this study was MiniSom (version 2.2.7) and the SOM model was trained using 10,000 interactions and a 10 × 10 grid on the training set with the learning rate of 0.05. For RF, we used 1000 trees, whereas for XGB, we chose the following hyperparameters: “binary: softmax” as the logistic function and “approx” as the tree method. The other parameters of the models were default. Features were normalized before being fed to LR, SVM, and SOM.

According to our evaluation strategy, for unsupervised models, we trained the models on the training set and found best thresholds on the validation set. For supervised models, we trained the models on the whole validation set. We then compared the performance of both supervised and unsupervised models on the test set. The accuracy, precision, recall, and F1 scores are calculated.

We also investigated the performance of the proposed model with fewer labels in comparison with that of the reference model. We randomly selected 200, 600, and 1000 labels in the validation set to find the thresholds for the unsupervised models and train the supervised models, and then test these on the whole test set. Each random selection is repeated 30 times, and then the mean and SD of the accuracy of the models are computed.

### Algorithm Application

We applied the designed SQA algorithm to 1144 cases of data collected in the HBO Department of PLAGH; each of the cases had a dynamic ECG record of nearly 24 hours. Each record of data was read by a clinical expert to give an overall signal quality evaluation result. According to the results, the data were divided into 3 groups, representing different grades of quality of the whole signals. We also scanned the data with an arrhythmia detection algorithm, which is commonly used in automatic dynamic ECG analysis, and the real-time alarm function of SensEcho. The core technology of the arrhythmia detection algorithm is traditional signal processing methods, including filtering and wavelet decomposition. We learned about the type, onset, and duration of each arrhythmia alarm detected by the arrhythmia detection algorithm. For the purpose of this study, a false alarm was defined as the onset of 1 arrhythmia alarm marked with poor signal quality. The proportion of different quality of signals, the number of various arrhythmia alarms, and the percentage of false alarms in each group were calculated.

## Results

### Model Performance

**Figure 5:**
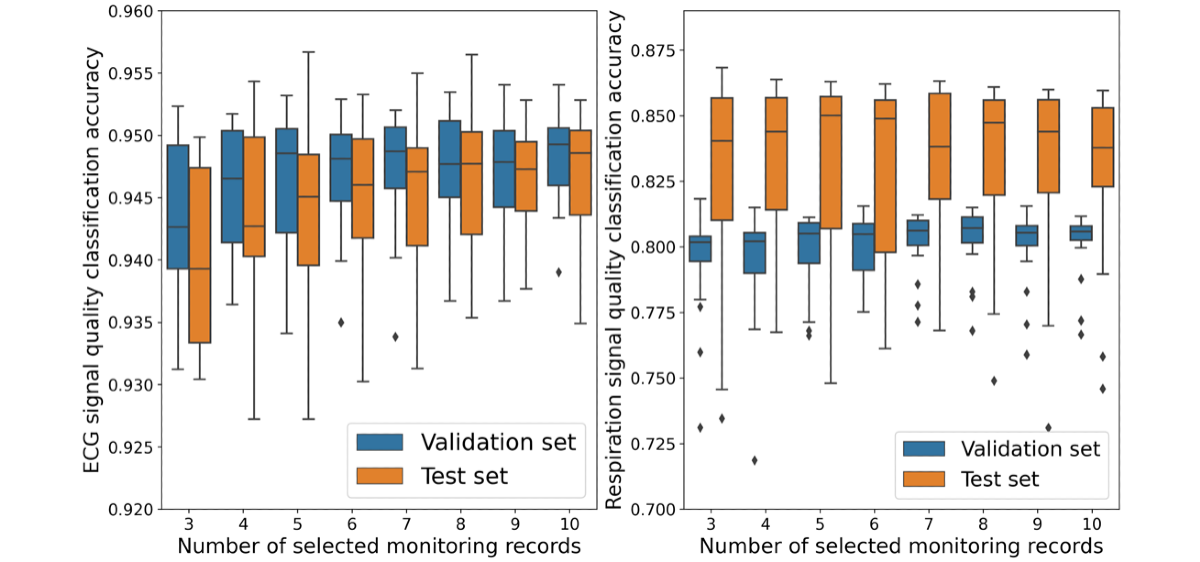
Quantitative evaluation of the model performance on the validation set and test set. ECG: electrocardiogram.

**Figure 6:**
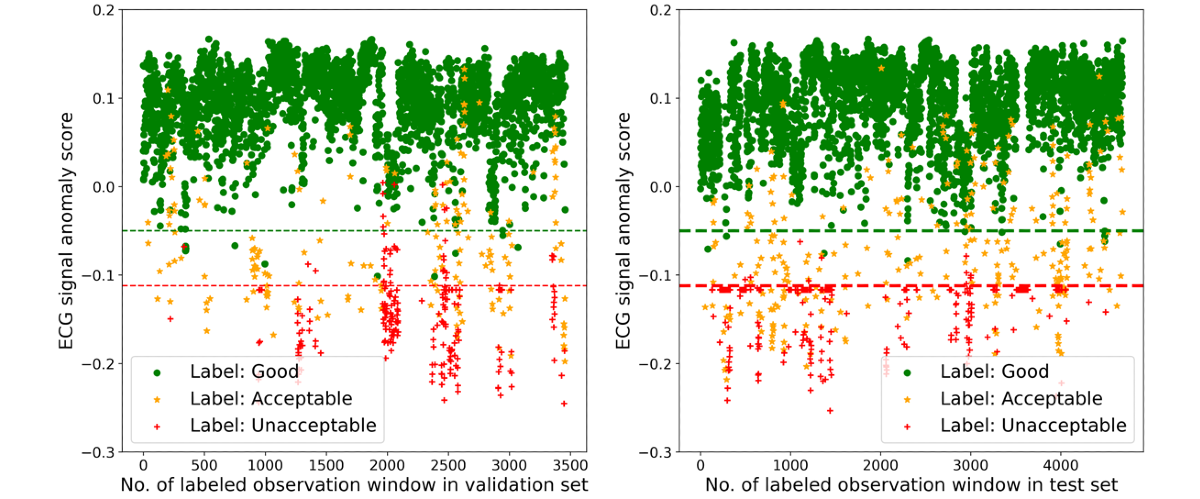
Electrocardiogram (ECG) signal anomaly scores on the validation set and test set, and the best performance thresholds.

**Figure 7:**
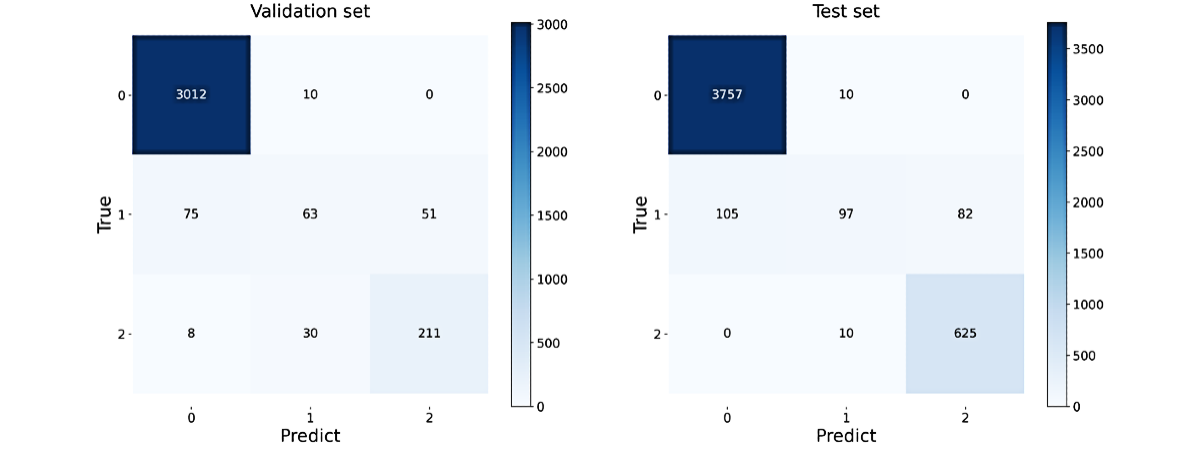
The electrocardiogram confusion matrixes of the results. 0: Good; 1: Acceptable; 2: Unacceptable.

**Figure 8:**
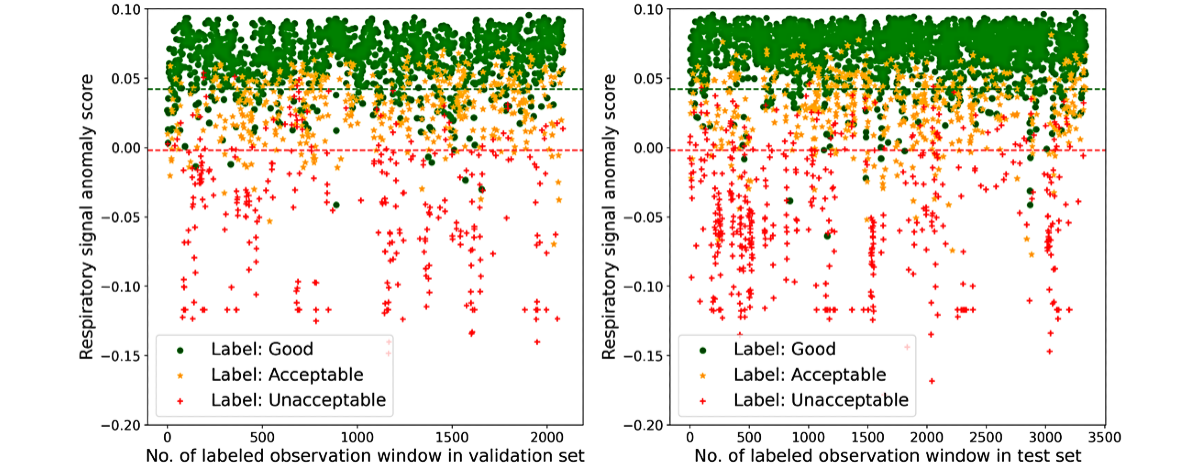
Respiratory signal anomaly scores on the validation set and test set, and the best performance thresholds.

**Figure 9:**
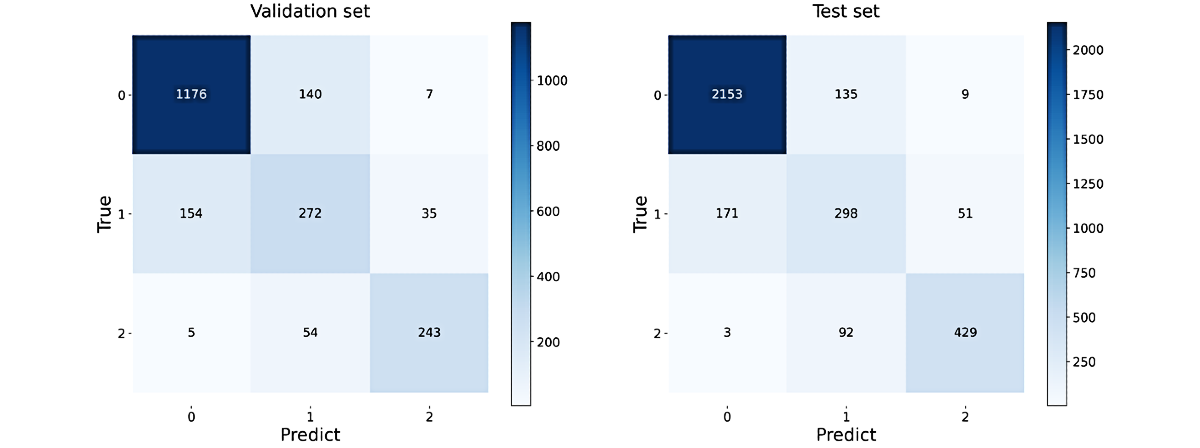
The respiratory confusion matrixes of the results. 0: Good; 1: Acceptable; 2: Unacceptable.

For the training set that is important for the IF model, we randomly selected monitoring records as described in the “Experiment Design” section and built the training sets to train the model to guarantee the variety and find the best performance of the model. Quantitative evaluation results of the model performance on the validation and test sets are shown in Figure 5. For ECG signals, the model performed at the same level on both validation and test sets, but for respiratory signals, the model performed slightly better on the test set than on the validation set. This is reasonable because the two data sets were constructed differently; thus, the test set was easier for SQA classification. Models that performed the best on the test set were selected for further study. The scores gained from the best model for ECG SQA and the best classification thresholds are shown in Figure 6, in which the accuracy reached 94.97% and 95.58% on the validation and test sets, respectively. The confusion matrixes are shown in Figure 7. Similarly, the scores for respiratory SQA and the thresholds are shown in Figure 8. This model achieved 81.06% and 86.20% accuracy on the validation and test sets, respectively. Figure 9 shows the confusion matrix of the results.

The results regarding the classification efficiency of the pathological ECG signal are summarized as follows: in the validation set, 100% (648/648) of good-grade and 23% (3/13) of acceptable-grade pathological ECG signals were classified correctly; however, 77% (10/13) of acceptable-grade signals were misclassified as good quality. In the test set, 99.8% (617/618) of good-grade and 31% (5/16) of acceptable-grade pathological signals were classified correctly; however, 1 sample of good-quality signal was misclassified as acceptable grade and 69% (11/16) of acceptable-grade signals were misclassified as good quality. The above results showed that the model also had a good classification effect on pathological signals: In this study, the vast majority of pathological signals were correctly classified and the misclassification will not increase false-negative decisions.

### Performance Evaluation Results

The classification results of the desired algorithm and reference models of the test set are summarized in Tables 3 and 4. From Table 3, it can be found that, for supervised models, the LR model performed the worst for both ECG and respiratory signals. Meanwhile, RF and XGB performed slightly better than the proposed algorithm. Understandably, supervised models generally have better performance than unsupervised models. For unsupervised models, SOM performed worse than the proposed model. For ECG SQA, the SOM achieved 0.91 accuracy and 0.55 F1 score on the validation set, indicating an insufficient generalization ability of the thresholds in this scenario for the model. We speculated that the complex pathological changes and noise in the data set made it difficult for SOM to perform dimensionality reduction and correctly map the model outputs to the SQA results. From Table 4, it can be found that the proposed model had a better performance when the number of labels is small. When the number of labels is greater than 1000, the performance of the supervised models was better than that of the proposed model. In other words, when we do not have enough labeled data, the unsupervised model is superior. However, we still recommend preparing slightly more labels as possible to guarantee the stability and generalization ability of the thresholds.

**Table 3:** Model performance on the test set.

Model		Electrocardiogram					Respiratory signal					
		Accuracy	Precision	Recall	F1	Accuracy		Precision	Recall	F1		
**Supervised models**												
	Logistic regression	0.79	0.79	0.61	0.50	0.79		0.72	0.55	0.59		
	Support vector machine	0.96	0.93	0.77	0.79	0.80		0.72	0.57	0.60		
	Random forest	0.97	0.95	0.84	0.87	0.92		0.88	0.85	0.87		
	Extreme gradient boosting	0.97	0.95	0.86	0.89	0.91		0.86	0.85	0.85		
**Unsupervised models**												
	Self-organizing maps	0.82	0.57	0.39	0.40	0.77		0.65	0.51	0.51		
	Isolation forest, proposed unsupervised model	0.96	0.90	0.77	0.80	0.86		0.79	0.78	0.78		

**Table 4:** The accuracy on the test set of models with fewer labeled data.

Number of labels			Logistic regression		Support vector machine		Random forest		Extreme gradient boosting		Self-organizing maps		Isolation forest, proposed unsupervised model
**ECG^a^, mean (SD)**													
	200	0.80 (0.00)		0.85 (0.05)		0.86 (0.06)		0.84 (0.06)		0.80 (0.01)		0.89 (0.06)	
	600	0.80 (0.00)		0.86 (0.05)		0.89 (0.06)		0.88 (0.05)		0.81 (0.01)		0.90 (0.06)	
	1000	0.81 (0.00)		0.90 (0.04)		0.93 (0.04)		0.92 (0.04)		0.81 (0.01)		0.93 (0.02)	
**Respiratory signal, mean (SD)**													
	200	0.71 (0.05)		0.71 (0.06)		0.80 (0.04)		0.79 (0.03)		0.70 (0.02)		0.82 (0.04)	
	600	0.75 (0.04)		0.75 (0.06)		0.85 (0.02)		0.84 (0.02)		0.73 (0.03)		0.84 (0.02)	
	1000	0.77 (0.04)		0.76 (0.06)		0.87 (0.01)		0.86 (0.01)		0.72 (0.07)		0.85 (0.01)	

^a^ECG: electrocardiogram.

### Case Validation

To further evaluate the performance of the algorithm on SQA, the algorithm was tested on several cases. In this paper, ECG and respiratory signals of a patient are illustrated. The patient is a 65-year-old male, standing 170 cm tall, and weighing 68 kg when admitted, and had been monitored by the SensEcho in the general ward of the HBO Department. He was diagnosed with coronary heart disease, posterior mitral valve prolapse, hypertension risk level 2, hyperuricemia, and fatty liver disease.

**Figure 10:**
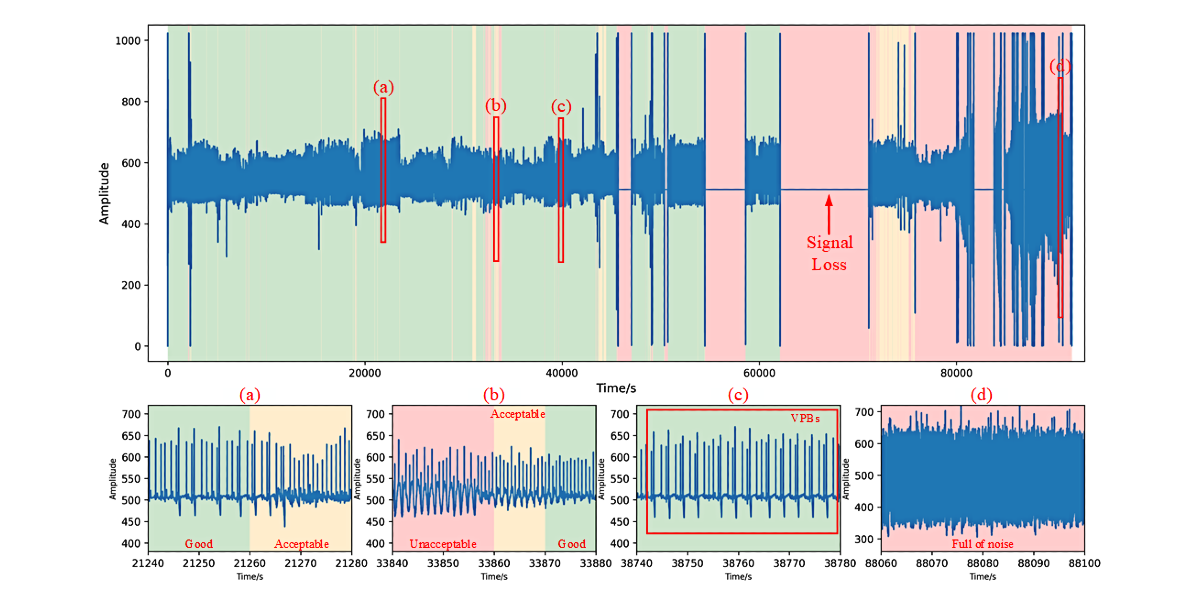
A signal quality assessment case example of the whole monitoring 24-hour electrocardiogram signal (Green: Good segments; Yellow: Acceptable segments; Red: Unacceptable segments).

**Figure 11:**
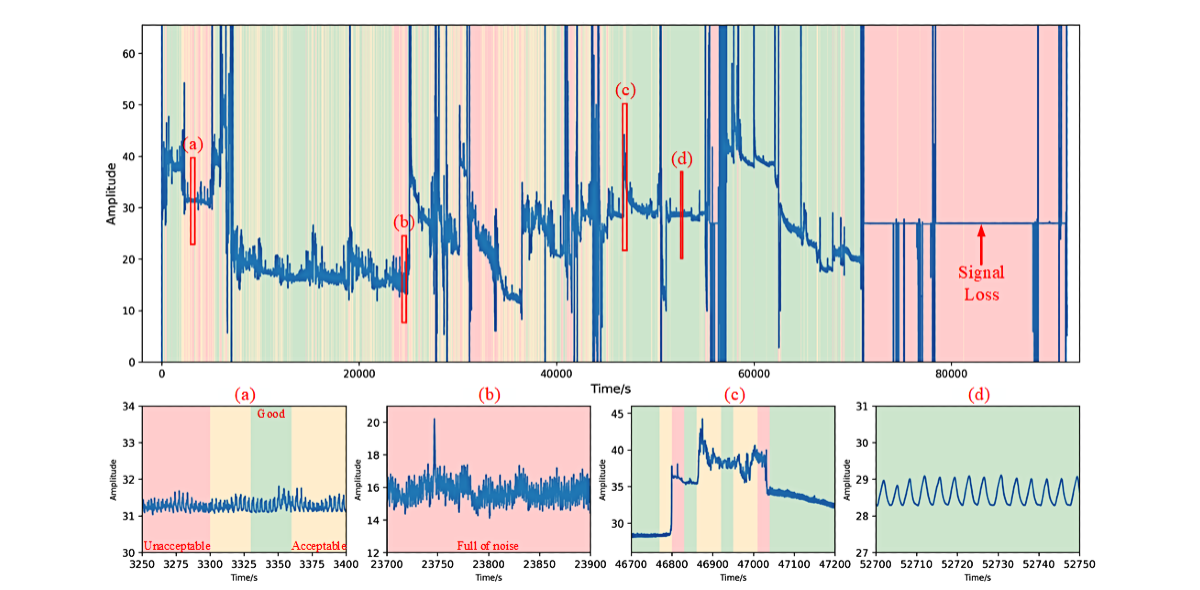
A signal quality assessment case example of the whole monitoring 24-hour respiratory signal (Green: Good segments; Yellow: Acceptable segments; Red: Unacceptable segments).

As shown in Figures 10 and 11, the different signal quality grades classified by the algorithm were marked in 3 colors: the green segments stand for the good quality, the yellow segments for the acceptable quality, and the red segments for the unacceptable quality. Furthermore, in these figures, 4 windows of the monitoring signals were selected to elaborate and illustrate the detailed signals and the classification results, respectively. It can be seen that the monitoring lasted for up to 24 hours, but there was not much high-quality data available in this case. Signal loss was the most common unacceptable signal quality expression and the segments were all marked in red. ECG and respiratory signals of the last few hours were full of noise, so it was suspected that the patient might have removed the device ahead of time.

We found that the pathological changes in ECG did not influence the SQA process directly (Figure 10). Most of the observation windows with ventricular premature beats (VPBs) were also marked in green and yellow correctly, that is, in this case the pathological changes were not filtered which met our expectations. In Figure 11, acceptable and unacceptable signal quality segments are more numerous and dispersed for respiratory signals compared with ECG signals. The good-quality segments were mainly concentrated during the patient’s bed rest period, as breath was more controllable and vulnerable to noise during the day. In conclusion, the algorithm demonstrated an excellent performance in this case and it can be used to automatically screen out the good-quality segments for further research.

### Algorithm Application Results

The algorithm application results are summarized in Table 5. The types of arrhythmia alarm we were concerned about were bradycardia, tachycardia, atrial premature beat (APB), VPB, atrial bigeminy, and atrial trigeminy. The “count” column represents the number of cases with a specific arrhythmia alarm detected; for example, bradycardia was detected in 525 cases out of the total 1144 cases. From Table 5, it can be seen that the age, weight, and height of the 3 groups of patients were basically on the same level, whereas the proportion of females increased in the medium and worst groups, indicating that the quality of ECG signal measured from female users might be poor due to hardware. The proportion of different signal quality grades in these cases means that the best group of patients has the highest percentage of good quality and the lowest percentage of unacceptable quality, whereas the worst group of patients has the lowest percentage of good quality and the highest percentage of unacceptable quality. Among these cases, the median [Q1-Q3] for good, acceptable, and unacceptable quality proportion was 90.0% [81.4%-95.9%], 4.8% [2.1%-8.0%], 4.0% [1.1%-9.3%], respectively. These results have 2 implications: First, the desired SQA algorithm is consistent with the common knowledge of people, which can be used to analyze the quality of signals measured by SensEcho automatically and quantitatively. Second, the vast majority of ECG signals measured by SensEcho are usable, which demonstrates that the wearable device can effectively monitor patients’ ECG signal for most of the time.

For the arrhythmia alarm results, ideally, the number of various arrhythmia alarms within each group should be similar. However, it was observed that the number of APBs and VPBs increased significantly (*P*=.02 and <.001, respectively), suggesting that the signal quality did affect the accuracy of the arrhythmia detection algorithm and that some of the alarms might have been caused by poor signal quality. For the defined false alarm results, the APBs and VPBs increased significantly (*P*<.001 for both) in the medium and worst groups, and the false alarm of VPBs even accounted for 60.4% [23.9%-87.3%] in the worst group, compared with 18.2% [0.0%-61.5%] for the VPBs among all cases. In addition, it was found that tachycardia had a very high false alarm proportion, probably due to the movement of patients with poor signal quality. We considered that the aforementioned types of false alarms can be detected and effectively reduced by the desired SQA algorithm. Meanwhile, it was also found that for some types of arrhythmia alarms such as those for atrial bigeminy and atrial trigeminy, the arrhythmia detection algorithm was accurate and rarely affected by the signal quality.

**Table 5:** Results of the SQA algorithm and the arrhythmia detection algorithm applied to the data collected from the Hyperbaric Oxygen Department.

Characteristic		Grouped by manual evaluation				Total (n=1144)		Count
		Best (n=671)	Medium (n=365)	Worst (n=108)				
**Demography**								
	Female, n (%)	239 (35.6)	145 (39.7)	45 (41.7)	429 (37.5)		1144	
	Age (year), median (Q1-Q3)	59.3 (53.0-66.8)	61.5 (54.0-67.9)	60.6 (53.5-66.7)	60.1 (53.5-67.1)		1144	
	Weight (kg), median (Q1-Q3)	70.0 (62.0-78.5)	71.0 (65.0-80.0)	70.0 (63.0-81.0)	70.3 (63.0-80.0)		1144	
	Height (cm), median (Q1-Q3)	168.0 (160.0-173.0)	168.0 (160.0-173.0)	167.5 (160.0-174.0)	168.0 (160.0-173.0)		1142	
**Proportion of different signal quality grades detected by the algorithm** (**%), median (Q1-Q3)**								
	Good	93.2 (87.0-97.3)	85.9 (78.6-92.0)	75.5 (61.9-86.2)	90.0 (81.4-95.9)		1141	
	Acceptable	3.8 (1.7-7.0)	5.8 (3.2-9.4)	5.8 (4.3-9.0)	4.8 (2.1-8.0)		1141	
	Unacceptable	2.1 (0.6-5.4)	7.0 (2.9-12.7)	15.2 (6.6-29.2)	4.0 (1.1-9.3)		1141	
**Arrhythmia alarm count, median (Q1-Q3)**								
	Bradycardia	4.0 (2.0-7.0)	3.0 (2.0-6.0)	2.0 (1.0-4.5)	4.0 (2.0-6.0)		525	
	Tachycardia	1.0 (1.0-2.0)	1.0 (1.0-2.0)	1.0 (1.0-2.0)	1.0 (1.0-2.0)		224	
	APB^a^	11.0 (4.0-34.5)	15.0 (6.0-42.0)	17.0 (4.0-46.0)	13.0 (5.0-39.0)		1103	
	VPB^b^	6.0 (2.0-25.2)	14.0 (4.0-54.0)	25.0 (5.0-76.0)	9.0 (3.0-39.0)		987	
	Atrial bigeminy	4.0 (1.0-13.0)	5.0 (2.0-9.0)	2.5 (2.0-6.0)	4.0 (2.0-10.0)		79	
	Atrial trigeminy	4.0 (2.0-10.5)	5.0 (1.0-10.8)	6.0 (2.2-11.2)	4.5 (1.8-11.0)		88	
**Defined false alarm proportion (%), median (Q1-Q3)**								
	Bradycardia	0.0 (0.0-0.0)	0.0 (0.0-0.0)	0.0 (0.0-0.0)	0.0 (0.0-0.0)		525	
	Tachycardia	50.0 (0.0-100.0)	100.0 (0.0-100.0)	100.0 (50.0-100.0)	100.0 (0.0-100.0)		224	
	APB	0.0 (0.0-9.9)	5.6 (0.0-28.6)	14.1 (0.0-70.6)	0.4 (0.0-19.1)		1103	
	VPB	6.2 (0.0-50.0)	35.6 (1.9-71.7)	60.4 (23.9-87.3)	18.2 (0.0-61.5)		987	
	Atrial bigeminy	0.0 (0.0-0.0)	0.0 (0.0-0.0)	0.0 (0.0-0.0)	0.0 (0.0-0.0)		79	
	Atrial trigeminy	0.0 (0.0-0.0)	0.0 (0.0-0.0)	0.0 (0.0-0.0)	0.0 (0.0-0.0)		88	

^a^APB: atrial premature beat.

^b^VPB: ventricular premature beat.

## Discussion

### Contributions and Principal Findings

Our highlights and key contributions are summarized as follows:

- We achieve the ECG and respiratory SQA by using an unsupervised model, IF, which has not been applied in SQA before. Furthermore, we attempted to verify the idea that the SQA process can be viewed as an anomaly detection. In this study, the proposed algorithm was superior than SOM and achieved moderate performance when compared with the supervised models.
- We applied the SQA algorithm to a large data set with 1144 records of ECG signal. The results demonstrate that the arrhythmia alarm accuracy could be influenced by the signal quality, and the SQA algorithm has the potential to reduce some specific types of arrhythmia false alarms such as tachycardia, APB, and VBP caused by poor signal quality.
- To our knowledge, this is one of the earliest studies that focuses on the quality of respiratory signals measured via the RIP technology. It provides a method to automatically select the high-quality segments of respiratory signal for further studies.

One featured point in our study is that 3 data sets that have different functions were used to construct and quantitatively validate the algorithm. In the workflow of our study, the training set was a large volume data set in which ideally all the patterns of the signal could be enumerated, while the validation set and the test set were unseen by the model when we trained it. We also conducted a very small experiment, where we directly trained the models on the validation set, found the best performance thresholds, and then evaluated the performance of the models on the test set. The results showed that for the ECG signal, the model achieved 0.92 accuracy and 0.72 F1 score, whereas for the respiratory signal, the model achieved 0.72 accuracy and 0.68 F1 score, which are lower than the current performance in the “Results” section. These results demonstrate that the diversity of patterns in the training set ensures the generalization performance of the unsupervised model. In fact, in an era of big data, it is easy to obtain a training set with a large sample size, yet lacking labels. The workflow we proposed in this study provides a feasible way to take advantage of the large sample size that can be applied in follow-up studies.

What should be emphasized is that we included the respiratory signal measured via RIP in this study for 2 reasons. First, the respiratory signal is an important physiological signal, which contains abundant personalized information, indicating the health status and disease deterioration of a person. More importantly, the quality of respiratory signal measured via RIP is not well investigated compared with ECG. In our study, we would like to point out that a signal with relatively little research and no fixed waveform could also be assessed by this method, which has the potential to be extended to other SQA scenarios such as impedance pneumography respiratory signal, dynamic blood pressure, and photoplethysmogram. That is, our study provides a practical workflow for other time-series physiological signal research groups to develop their own applicative SQA algorithms.

### Limitations

There are also some limitations to our work. First, the model we used was an unsupervised machine learning model, which lacks enough interpretability and the performance is largely determined by the quality of the training set. We attempted several construction methods of the training set, yet it was hard to guarantee that the models achieved the best performance. Second, the classification results of the models for the medium grade of signal quality were not good. The sensitivities of the algorithm for this grade are only 0.34 for ECG and 0.57 for respiratory signals, respectively, which seriously lower the overall F1 scores of the models. This is because the medium level of signal quality is always the hardest to classify even manually. We tried some approaches such as data augmentation and constructing an artificial training set. However, the results showed no significant improvement. It is worth mentioning that the SOM showed moderate performance in the unsupervised methods, perhaps because, in our study, the framework, especially the training and generalization methods, was not suitable for this model. Further to this point, SOM and the rapidly evolving deep learning methods are worth being investigated after further accumulation of data. Third, as the validation of the algorithm on pathological signals was insufficient, although the results in this study were good, we still consider that the algorithm has the risk of misclassifying pathological changes as abnormal as a result of noise. We thus need to further validate the algorithm, which demands more pathological data accumulation and long-term feedback of actual use from clinicians.

### Future Work

Our future research includes the following. First, the algorithm calls for more comprehensive experimental validation. Accordingly, we should further verify the performance of the model in the presence of pathological changes and quantify how much the model can reduce the false alarm rate. It requires long-term usage and more data collection, especially from patients with specific diseases such as arrhythmia and chronic obstructive pulmonary disease. Second, we will test the time usage and real-time performance of the algorithm. To our knowledge, the IF model operation does not take too much time when the thresholds are determined, yet the feature extraction process is more time-consuming. As we preliminarily tested, the whole SQA process for ECG signal takes 0.3-0.5 seconds on server for every observation window (10 seconds). For respiratory signal, it takes less than 0.1 seconds for every observation window (30 seconds). We will integrate the algorithm into the server to achieve the real-time SQA. Third, there are many mHealth and uHealth apps nowadays, but there is a lack of assessment of the data measured under nonlaboratory conditions and their usability. Based on the algorithm we developed, we will further evaluate the value of the wearable device, SensEcho, in daily life situations from a signal quality perspective, find the cause of the decrease in signal quality, and improve the both hardware and software of the wearable device. We believe that this will further promote the application of mHealth and uHealth.

### Conclusions

In this study, the results verified our hypothesis that the SQA problem can be seen as an anomaly detection. We built a model based on the unsupervised machine learning model, IF, to avoid heavy data annotation work and to realize ECG and respiratory SQA. What distinguishes us from other studies that used the IF model is that we used a small amount of labeled data to enable the mapping of model scores to human cognitive classification results. Our validation results indicate that the proposed algorithm is superior than SOM and shows a moderate performance compared with supervised models. Meanwhile, the proposed algorithm has the advantages of flexibility, easy adjustment, and better performance with few labeled data. In addition, the pathological changes in our case are correctly classified, demonstrating the model’s good application effect. The algorithm application results on 1144 cases from the clinic suggest that the proposed algorithm has the potential to reduce some types of arrhythmia false alarms such as tachycardia, APB, and VBP.

Middle-aged and elderly people, such as patients in the HBO Department in this study, often suffer from complex chronic diseases and are at relatively high risk even in hospitals. Therefore, the adoption of wearable devices in clinics and the advancement of data analysis could provide easily accessible health care that can greatly benefit this population. We consider that the proposed algorithm can advance the clinical apps of wearable devices and facilitate follow-up mHealth and uHealth studies of various time-series physiological signals.

## Abbreviations

APB: atrial premature beat
CFDA: China Food and Drug Administration
ECG: electrocardiogram
HBO: hyperbaric oxygen
IF: isolation forest
LR: logistic regression
mHealth: mobile health
MITDB: MIT-BIH Arrhythmia Database
PLAGH: Chinese PLA General Hospital
RF: random forest
RIP: respiratory inductive plethysmography
SOM: self-organizing maps
SQA: signal quality assessment
SQIs: signal quality indices
SVM: support vector machine
uHealth: ubiquitous health
VPB: ventricular premature beat
XGB: extreme gradient boosting

## References

[1] Ernsting C, Stühmann LM, Dombrowski SU, Voigt-Antons J, Kuhlmey A, Gellert P (2019). Associations of Health App Use and Perceived Effectiveness in People With Cardiovascular Diseases and Diabetes: Population-Based Survey. JMIR Mhealth Uhealth.

[2] Yang Q, Van Stee SK (2019). The Comparative Effectiveness of Mobile Phone Interventions in Improving Health Outcomes: Meta-Analytic Review. JMIR Mhealth Uhealth.

[3] World Health Organization (2011). mHealth: new horizons for health through mobile technologies.

[4] Baig MM, GholamHosseini H, Moqeem AA, Mirza F, Lindén M (2017). A Systematic Review of Wearable Patient Monitoring Systems - Current Challenges and Opportunities for Clinical Adoption. J Med Syst.

[5] Patel S, Park H, Bonato P, Chan L, Rodgers M (2012). A review of wearable sensors and systems with application in rehabilitation. J Neuroeng Rehabil.

[6] Weenk M, van Goor H, Frietman B, Engelen LJ, van Laarhoven CJ, Smit J, Bredie SJ, van de Belt TH (2017). Continuous Monitoring of Vital Signs Using Wearable Devices on the General Ward: Pilot Study. JMIR Mhealth Uhealth.

[7] Kamei T, Kanamori T, Yamamoto Y, Edirippulige S (2020). The use of wearable devices in chronic disease management to enhance adherence and improve telehealth outcomes: A systematic review and meta-analysis. J Telemed Telecare.

[8] Ludikhuize J, Smorenburg SM, de RSE, de JE (2012). Identification of deteriorating patients on general wards; measurement of vital parameters and potential effectiveness of the Modified Early Warning Score. J Crit Care.

[9] Churpek MM, Yuen TC, Edelson DP (2013). Predicting clinical deterioration in the hospital: the impact of outcome selection. Resuscitation.

[10] Gambarotta N, Aletti F, Baselli G, Ferrario M (2016). A review of methods for the signal quality assessment to improve reliability of heart rate and blood pressures derived parameters. Med Biol Eng Comput.

[11] Bizzego A, Gabrieli G, Furlanello C, Esposito G (2020). Comparison of Wearable and Clinical Devices for Acquisition of Peripheral Nervous System Signals. Sensors (Basel).

[12] Satija U, Ramkumar B, Manikandan MS (2018). A Review of Signal Processing Techniques for Electrocardiogram Signal Quality Assessment. IEEE Rev Biomed Eng.

[13] Liu F, Wei S, Lin F, Jiang X, Liu C, Liu C, Li J (2020). An Overview of Signal Quality Indices on Dynamic ECG Signal Quality Assessment. Feature Engineering and Computational Intelligence in ECG Monitoring.

[14] Langley P, Marco L, King S, Duncan D, Murray A (2012). An algorithm for assessment of quality of ECGs acquired via mobile telephones. Computing in Cardiology.

[15] Johannesen L (2011). Assessment of ECG quality on an Android platform Computing in Cardiology.

[16] Zhang Y, Wei S, Zhang L, Liu C (2018). Comparing the Performance of Random Forest, SVM and Their Variants for ECG Quality Assessment Combined with Nonlinear Features. J. Med. Biol. Eng.

[17] Li Q, Mark RG, Clifford GD (2008). Robust heart rate estimation from multiple asynchronous noisy sources using signal quality indices and a Kalman filter. Physiol Meas.

[18] Clifford G, Lopez D, Li Q, Rezek I (2011). Signal quality indices and data fusion for determining acceptability of electrocardiograms collected in noisy ambulatory environments. Computing in Cardiology.

[19] Behar J, Oster J, Li Q, Clifford G (2012). A single channel ECG quality metric. Computing in Cardiology.

[20] Ansari S, Gryak J, Najarian K (2018). oise Detection in Electrocardiography Signal for Robust Heart Rate Variability Analysis: A Deep Learning Approach.

[21] Shi Y, Han N, Li P, Yang Z, Yuan Q, Du Y (2019). Robust Assessment of ECG Signal Quality for Wearable Devices.

[22] Satija U, Ramkumar B, Manikandan MS (2018). An automated ECG signal quality assessment method for unsupervised diagnostic systems. Biocybernetics and Biomedical Engineering.

[23] Zhao Z, Liu C, Li Y, Li Y, Wang J, Lin B, Li J (2019). Noise Rejection for Wearable ECGs Using Modified Frequency Slice Wavelet Transform and Convolutional Neural Networks. IEEE Access.

[24] Charlton PH, Bonnici T, Tarassenko L, Clifton DA, Beale R, Watkinson PJ, Alastruey J (2021). An impedance pneumography signal quality index: Design, assessment and application to respiratory rate monitoring. Biomed Signal Process Control.

[25] Steinberg C, Philippon F, Sanchez M, Fortier-Poisson P, O'Hara G, Molin F, Sarrazin J, Nault I, Blier L, Roy K, Plourde B, Champagne J (2019). A Novel Wearable Device for Continuous Ambulatory ECG Recording: Proof of Concept and Assessment of Signal Quality. Biosensors (Basel).

[26] Liu C, Zhang X, Zhao L, Liu F, Chen X, Yao Y, Li J (2019). Signal Quality Assessment and Lightweight QRS Detection for Wearable ECG SmartVest System. IEEE Internet Things J.

[27] Li L (2016). A Quality Assessment Method of Single-Lead ECG Signal Based on Spectral Analysis.

[28] Cao D, Li D, Zhang Z, Liu X, Liang H, He M, Yu M (2019). [Design and preliminary validation of a ubiquitous and wearable physiological monitoring system]. Sheng Wu Yi Xue Gong Cheng Xue Za Zhi.

[29] Xu H, Li P, Yang Z, Liu X, Wang Z, Yan W, He M, Chu W, She Y, Li Y, Cao D, Yan M, Zhang Z (2020). Construction and Application of a Medical-Grade Wireless Monitoring System for Physiological Signals at General Wards. J Med Syst.

[30] Li Q, Clifford GD (2012). Signal quality and data fusion for false alarm reduction in the intensive care unit. J Electrocardiol.

[31] Xia H, Garcia G, Mcbride J, Sullivan A, Zhao X (2011). Computer algorithms for evaluating the quality of ECGs in real time.

[32] Naseri H, Homaeinezhad M (2015). Electrocardiogram signal quality assessment using an artificially reconstructed target lead. Comput Methods Biomech Biomed Engin.

[33] Behar J, Oster J, Clifford GD, Qiao Li (2013). ECG Signal Quality During Arrhythmia and Its Application to False Alarm Reduction. IEEE Trans. Biomed. Eng.

[34] Kuzilek J, Huptych M, Chudacek V, Spilka J, Lhotska L (2011). Data driven approach to ECG signal quality assessment using multistep SVM classification. Computing in Cardiology.

[35] Zhang Y, Wei S, Long Y, Liu C (2015). Performance Analysis of Multiscale Entropy for the Assessment of ECG Signal Quality. Journal of Electrical and Computer Engineering.

[36] Elghazzawi Z (1999). Method and apparatus for removing artifact from physiological signals. European Patent EP19980308473.

[37] Friesen G, Jannett T, Jadallah M, Yates S, Quint S, Nagle H (1990). A comparison of the noise sensitivity of nine QRS detection algorithms. IEEE Trans Biomed Eng.

[38] Chen Y, Wu W (2018). Isolation Forest as an Alternative Data-Driven Mineral Prospectivity Mapping Method with a Higher Data-Processing Efficiency. Nat Resour Res.

[39] Ding Z, Fei M (2013). An Anomaly Detection Approach Based on Isolation Forest Algorithm for Streaming Data using Sliding Window. IFAC Proceedings Volumes.

[40] Liu F, Ting K, Zhou Z (2008). Isolation Forest.

[41] Meinard Müller (2007). Dynamic time warping. Information retrieval for music and motion.

[42] Zhang G, Kinsner W, Huang B (2009). Electrocardiogram data mining based on frame classification by dynamic time warping matching. Comput Methods Biomech Biomed Engin.

[43] Liu C, Zhang X, Zhao L, Liu F, Chen X, Yao Y, Li J (2019). Signal Quality Assessment and Lightweight QRS Detection for Wearable ECG SmartVest System. IEEE Internet Things J.

[44] Kohonen T (1997). The Basic SOM. Self-Organizing Maps.

[45] Roy M, Gupta R, Sharma K (2020). Photoplethysmogram Signal Quality Evaluation by Unsupervised Learning Approach. Photoplethysmogram Signal Quality Evaluation by Unsupervised Learning Approach.

[46] Ghosal P, Gupta R (2015). Classification of photoplethysmogram signal using self organizing map. Classification of photoplethysmogram signal using self organizing map.

[47] Mehmood Y, Abbas M, Chen X, Honkela T (2011). Self-Organizing Maps of Nutrition, Lifestyle and Health Situation in the World.

[48] Tibaduiza DA, Mujica LE, Rodellar J (2012). Damage classification in structural health monitoring using principal component analysis and self-organizing maps. Struct. Control Health Monit.

